# Supplementary material for: Behavioral differences among domestic cats in the response to cat-attracting plants and their volatile compounds reveal a potential distinct mechanism of action for actinidine
Source: BMC Biol. 2022 Aug 25;20:192. doi: 10.1186/s12915-022-01369-1 (PMC9414117; doi:10.1186/s12915-022-01369-1)
Supplement: Supplementary file 1 — Additional file 1: Figures S1–S14. [file 12915_2022_1369_MOESM1_ESM.docx]

**Behavioral differences among domestic cats in the response to cat-attracting plants and their volatile compounds reveal a potential distinct mechanism of action for actinidine**

Sebastiaan Bol, Adrian Scaffidi, Evelien M. Bunnik, Gavin R. Flematti

BMC Biology, 2022

**Figures S1 – S14**


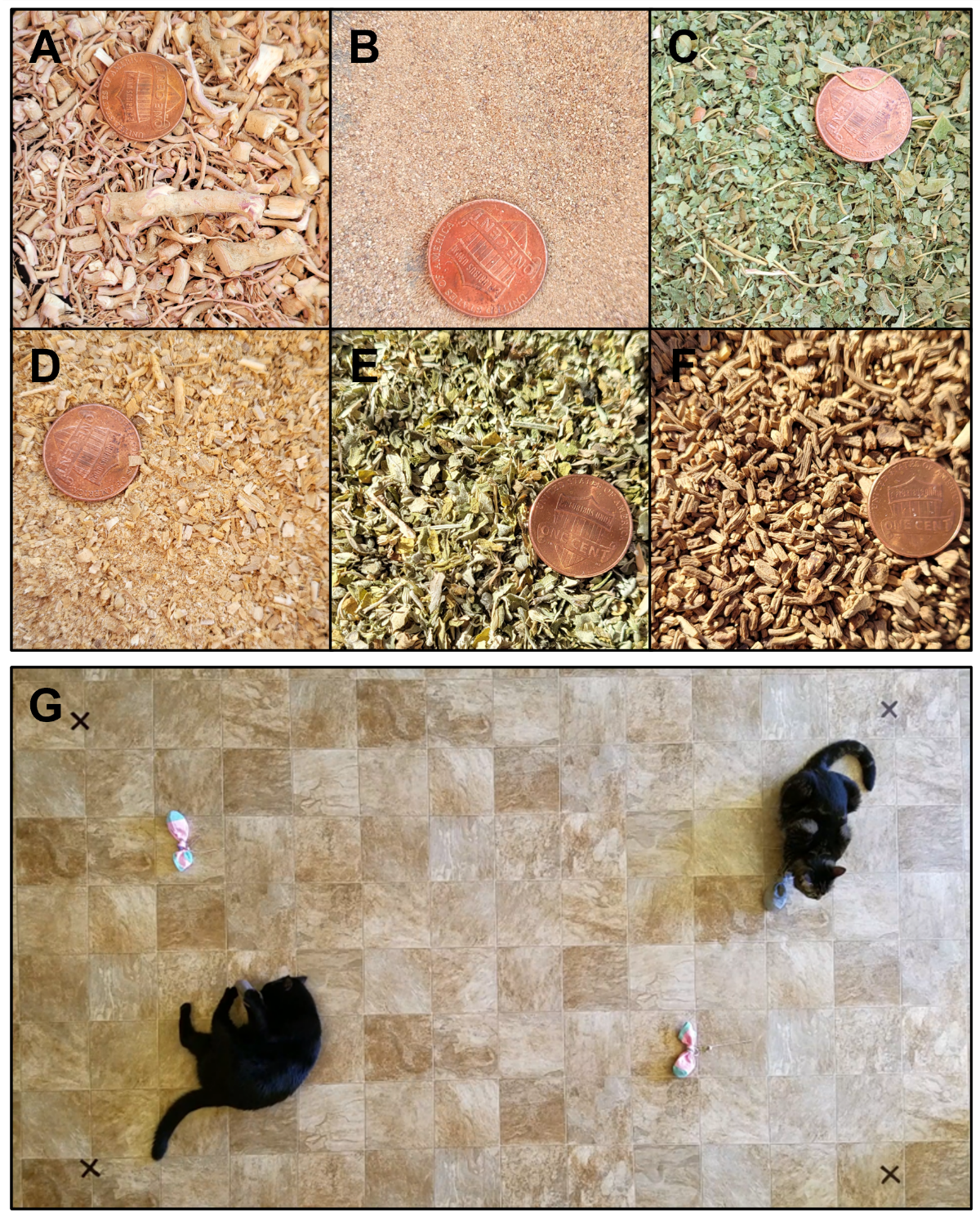


**Figure S1: Plant materials used and the testing area.** Top (**A – F**): Close-up photographs of the plant materials used in the study: (**A**) lyophilized and cut *Acalypha indica* or Indian nettle root, (**B**) dried, powdered *Actinidia polygama* or silver vine fruit gall, (**C**) dried and cut Texas-grown *A. polygama* leaves, (**D**) *Lonicera tatarica* or Tatarian honeysuckle sawdust, (**E**) dried and cut *Nepeta cataria* or catnip leaves, and (**F**) dried and cut *Valeriana officinalis* or valerian root. A United States penny (19 mm diameter) is used as a size reference. Bottom (**G**): The testing area with 4 mounted socks. The black x’s served to assure the relevant area of the testing area was being captured by the camera.


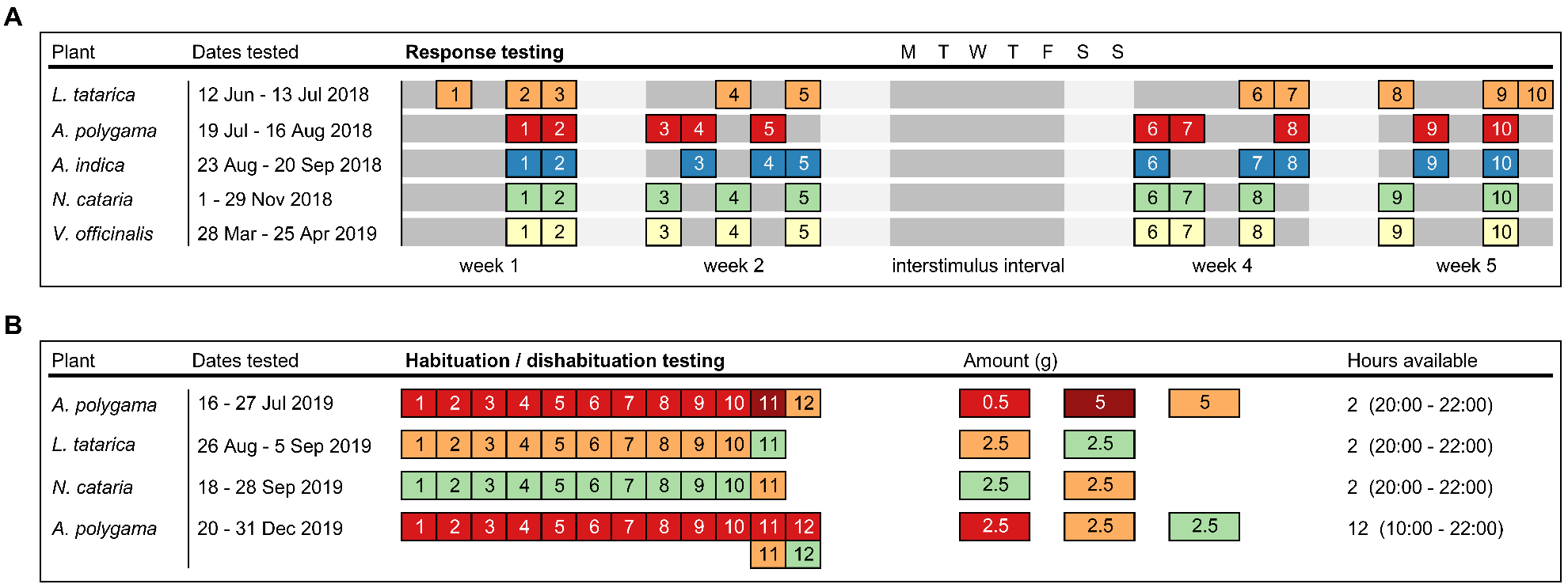


**Figure S2.** Timeline for testing cat-attracting plants. (**A**) Each cat-attracting plant was tested on 10 different days (no. 1 – 10), 10 hours per day, to learn more about response duration, response frequency, and behavior during the responses. The tests were done in two periods of two weeks, separated by an interstimulus interval of at least 9 days. Testing was done 2 – 3 days per week. There was always at least one week between testing the different plants (see “Dates tested”). (**B**) Three different cat-attracting plants were offered for 10 consecutive days (2 or 12 hours per day) to test for habituation. After 10 days the cats were offered a different plant to test dishabituation. Plant materials are color-coded according to the color scheme used in panel A.


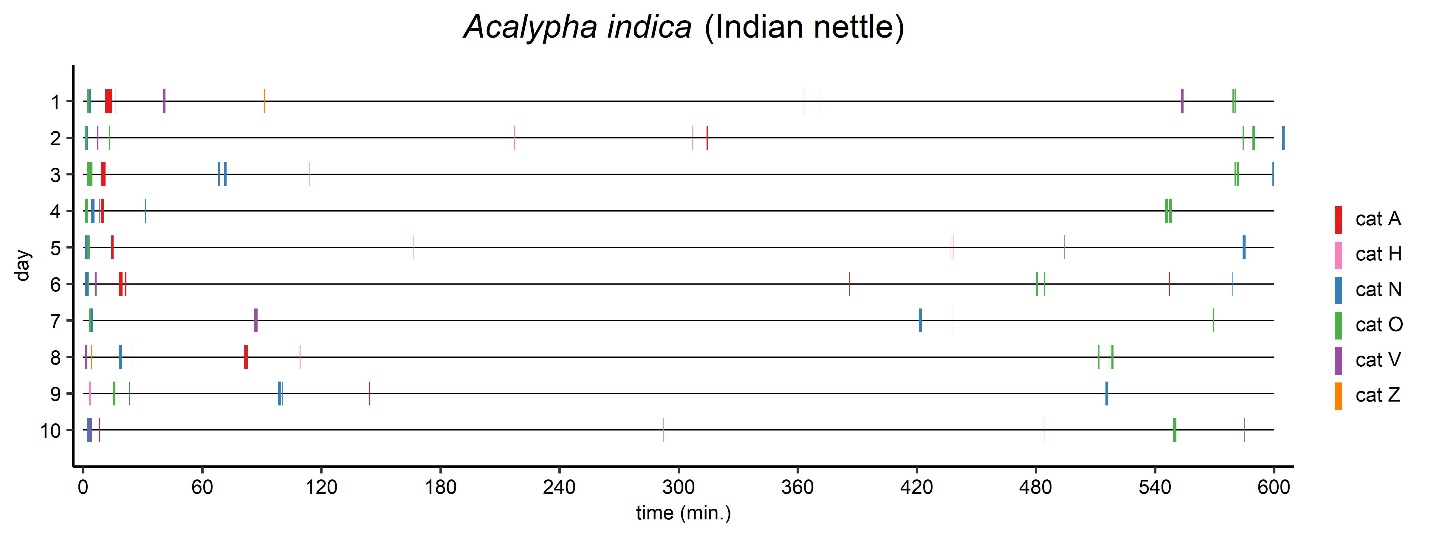

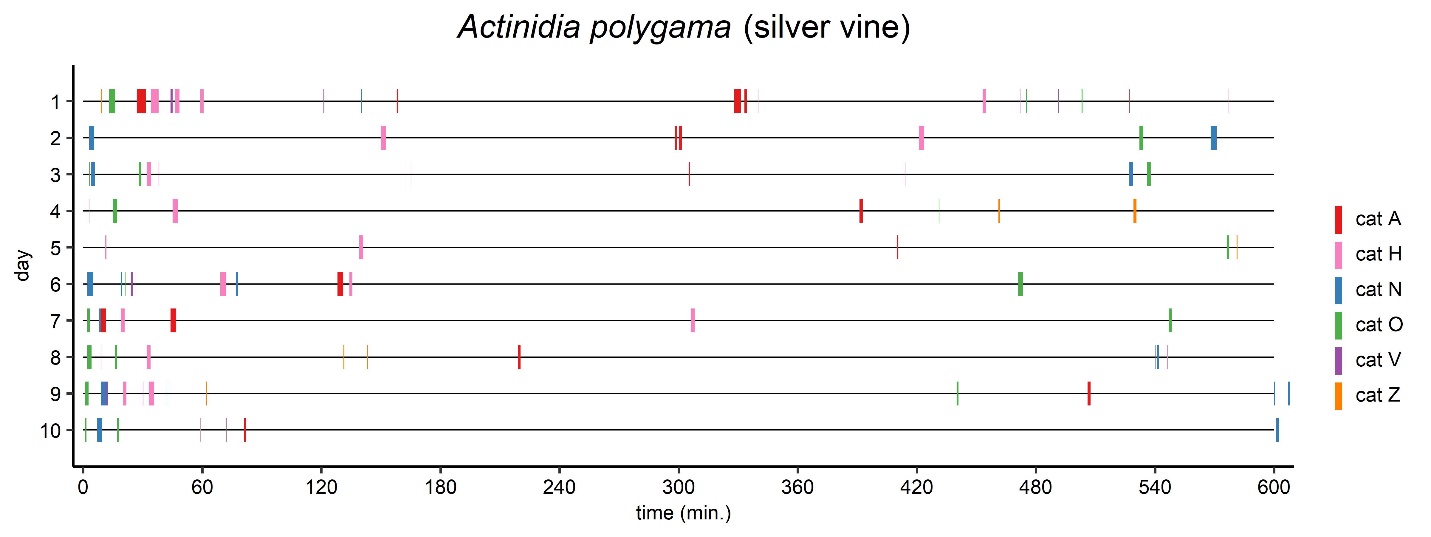

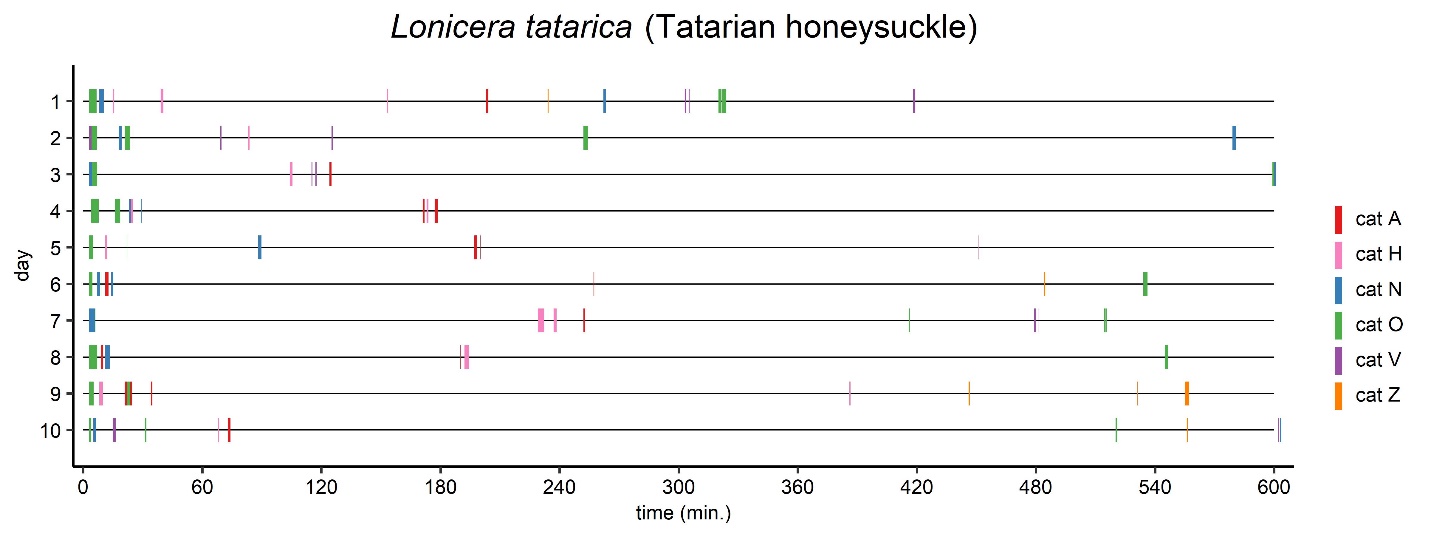

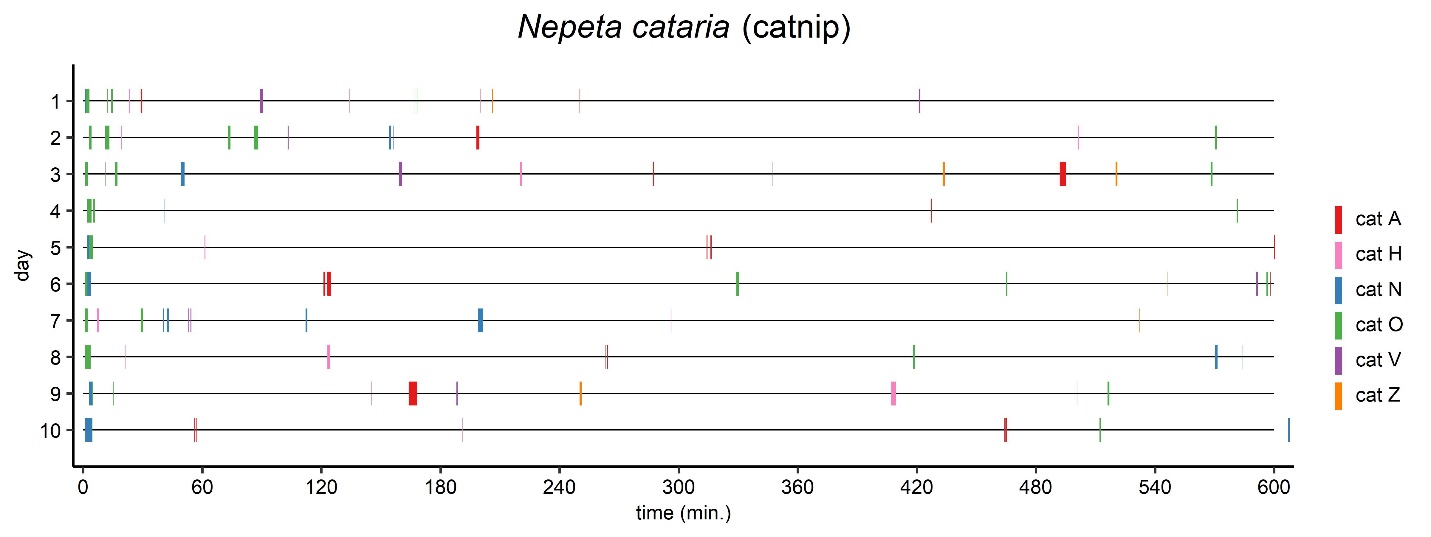

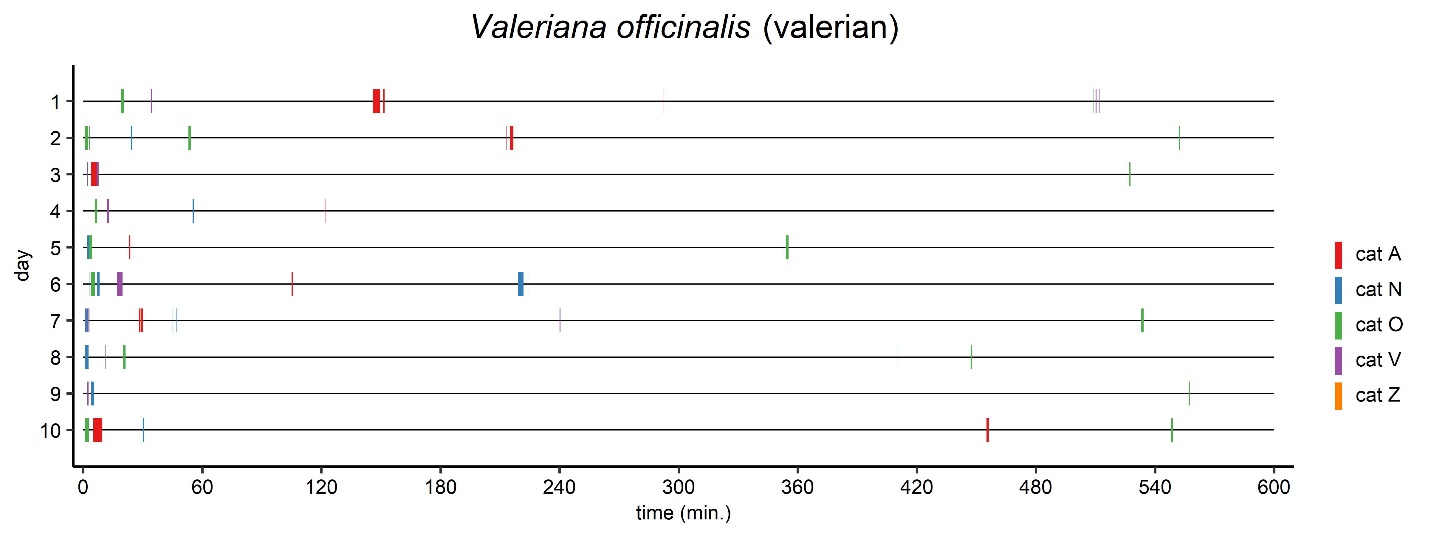


**Figure S3.** Graphical overview showing time of day of the responses, response frequency and response duration for the 6 domestic cats to *A. indica*, *A. polygama*, *L. tatarica*, *N. cataria*, and *V. officinalis*. Each plant was available on 10 days for 10 hours (600 minutes), between 9:30 and 19:30. Responses that lasted only a couple of seconds sometimes do not show.


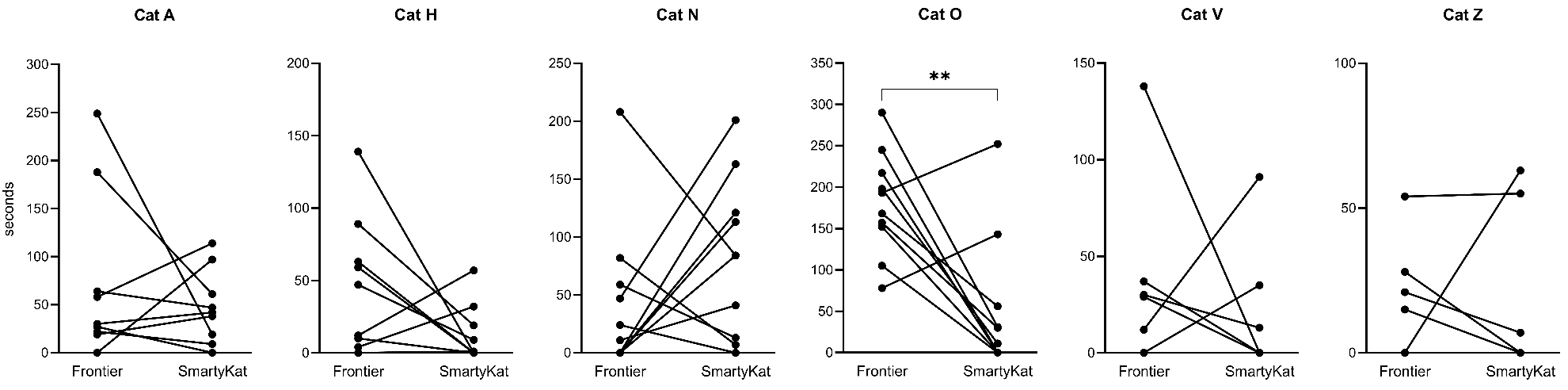


**Figure S4.** Response duration of 6 domestic cats to two different sources of *N. cataria*. Each dot represents the total response duration on one of the 10 testing days. The median response duration of cat O to catnip from Frontier was significantly longer than the median response duration to catnip from SmartyKat (P = 0.0098, Wilcoxon matched-pairs signed rank test). ** P < 0.01


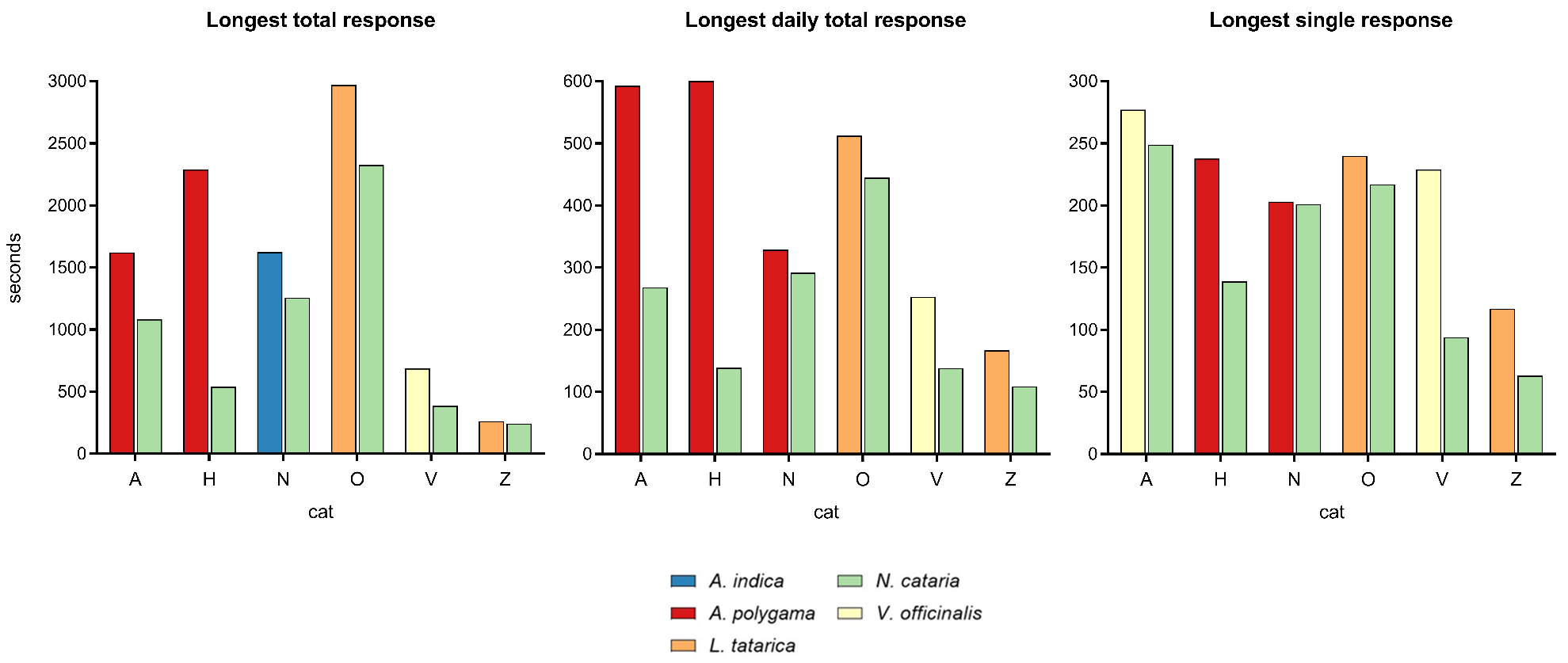


**Figure S5.** The longest total response (100 hours availability), the longest daily response (10 hours availability), and the longest single response for each cat, compared to *N. cataria* (catnip).


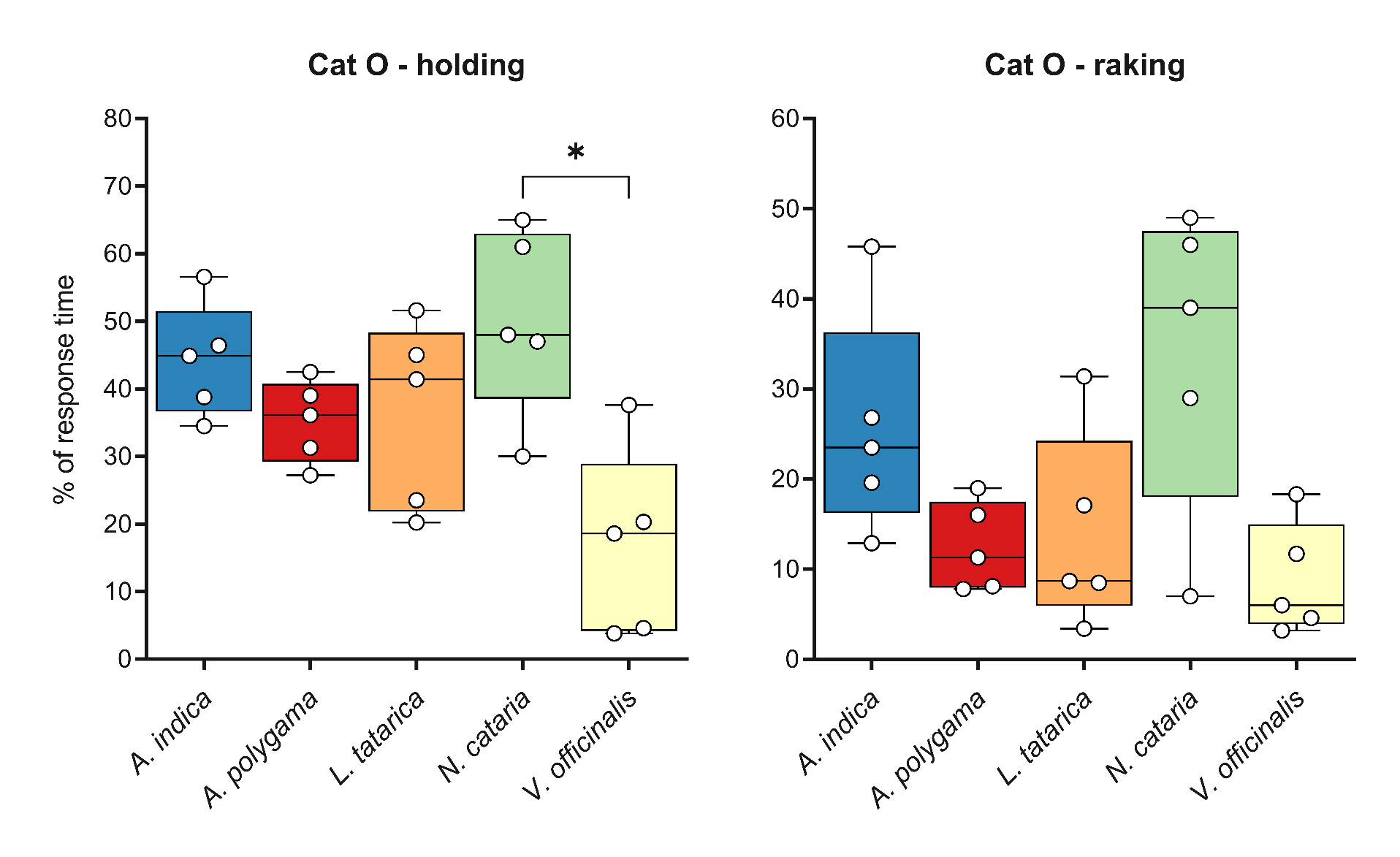


**Figure S6.** Box and whisker plots showing the time spent holding and raking by cat O in response to 5 cat-attracting plant species. Data from 5 responses nearest to 60 seconds are shown for each plant. Time is expressed as the percentage of the total response duration. This is a different representation of the data shown in **Figure 8** (cat O). The differences between the plants for both holding and raking were statistically significant (Kruskal-Wallis, P < 0.05). The P value shown is from Dunn’s post-hoc test. * P < 0.05


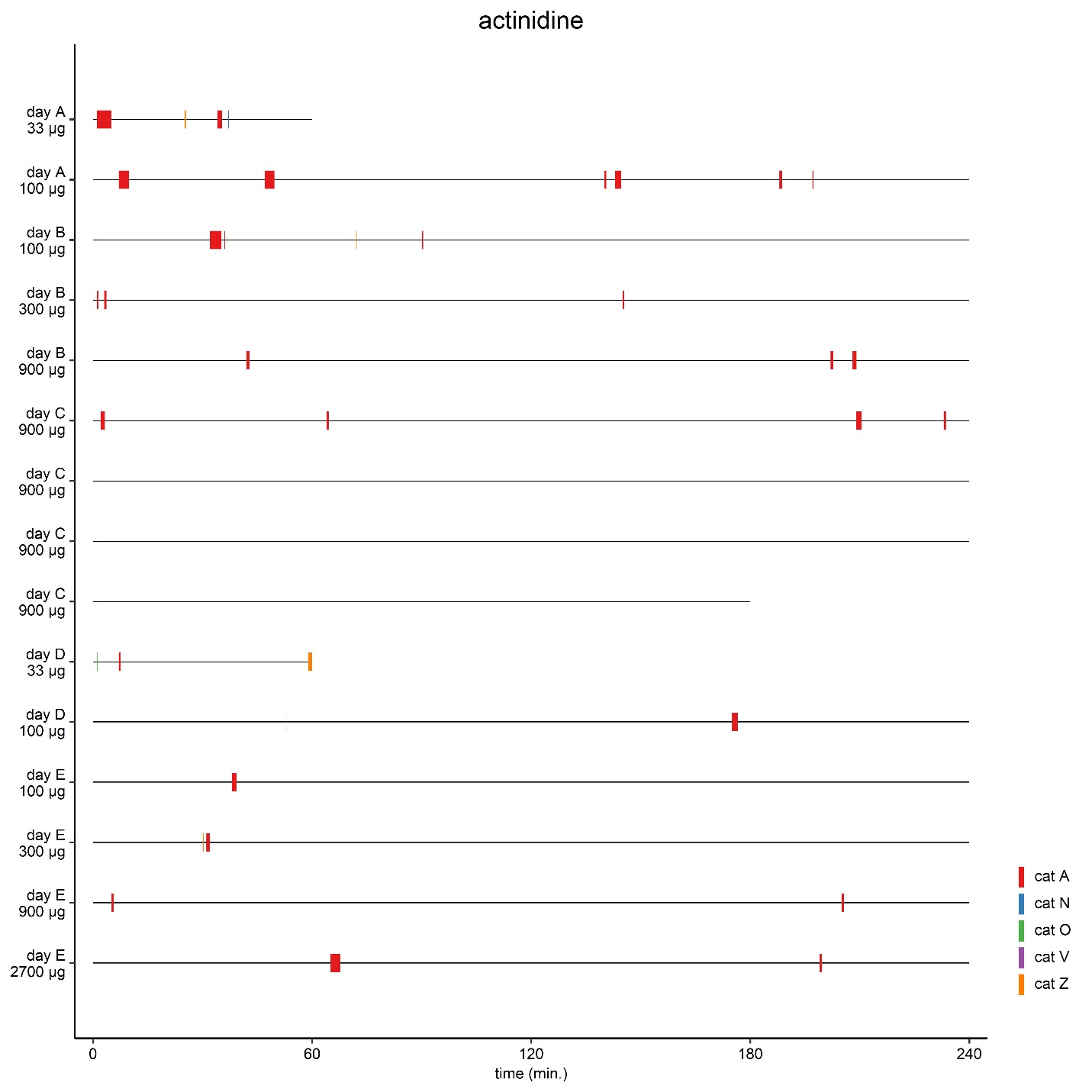

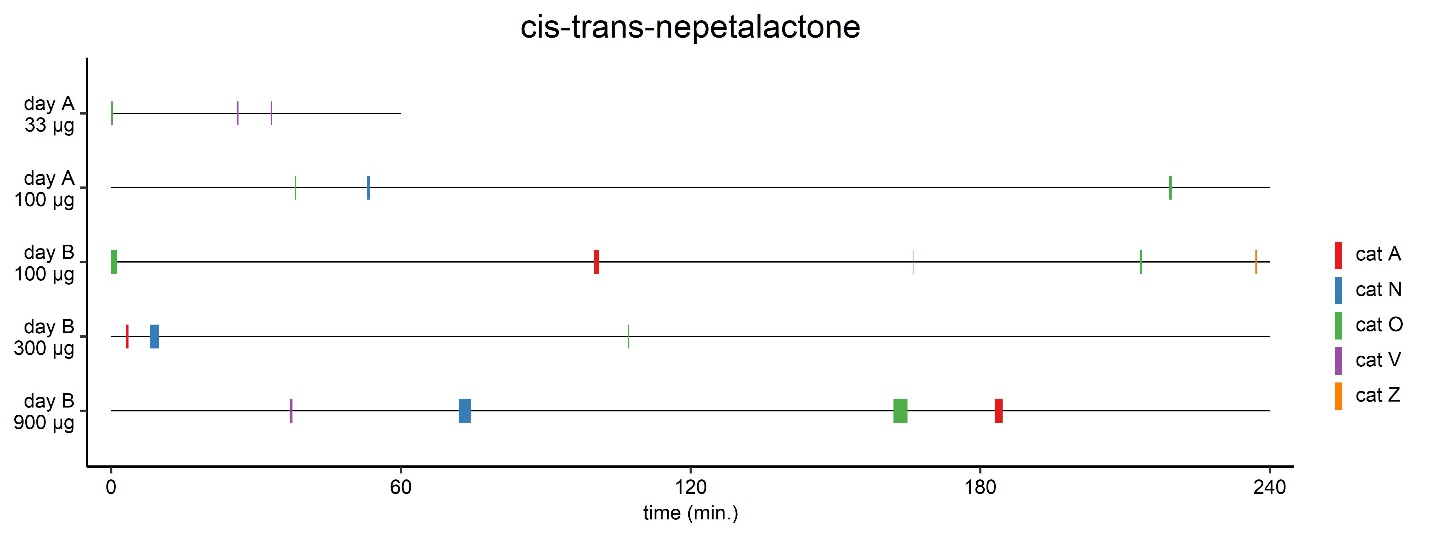

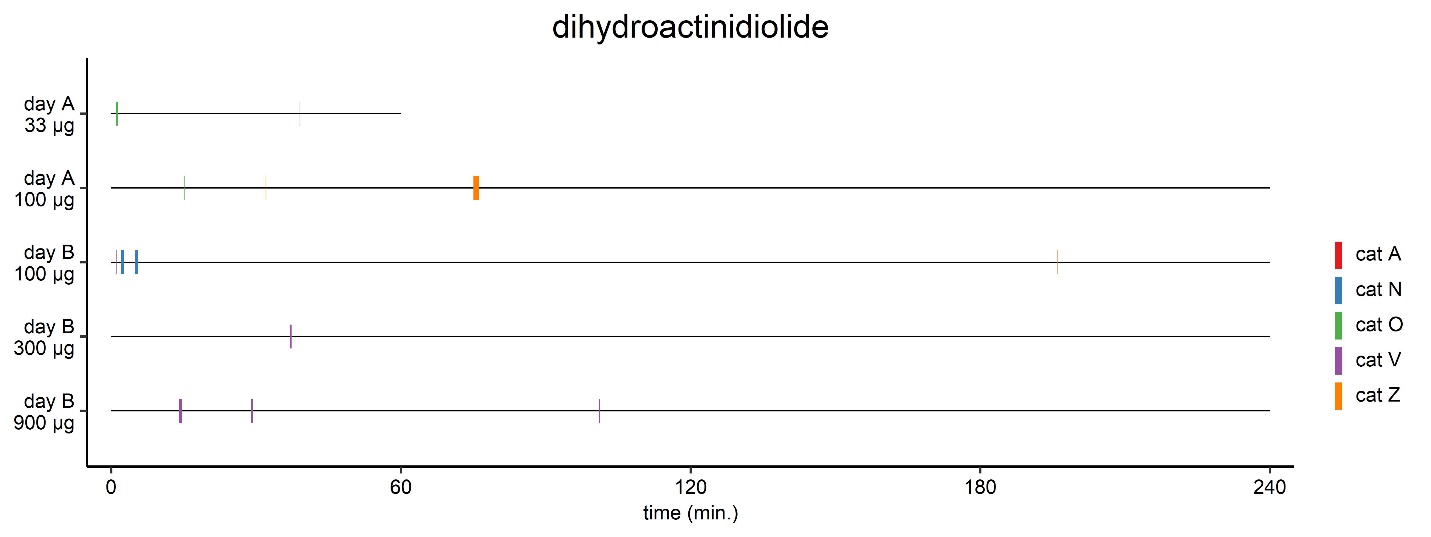

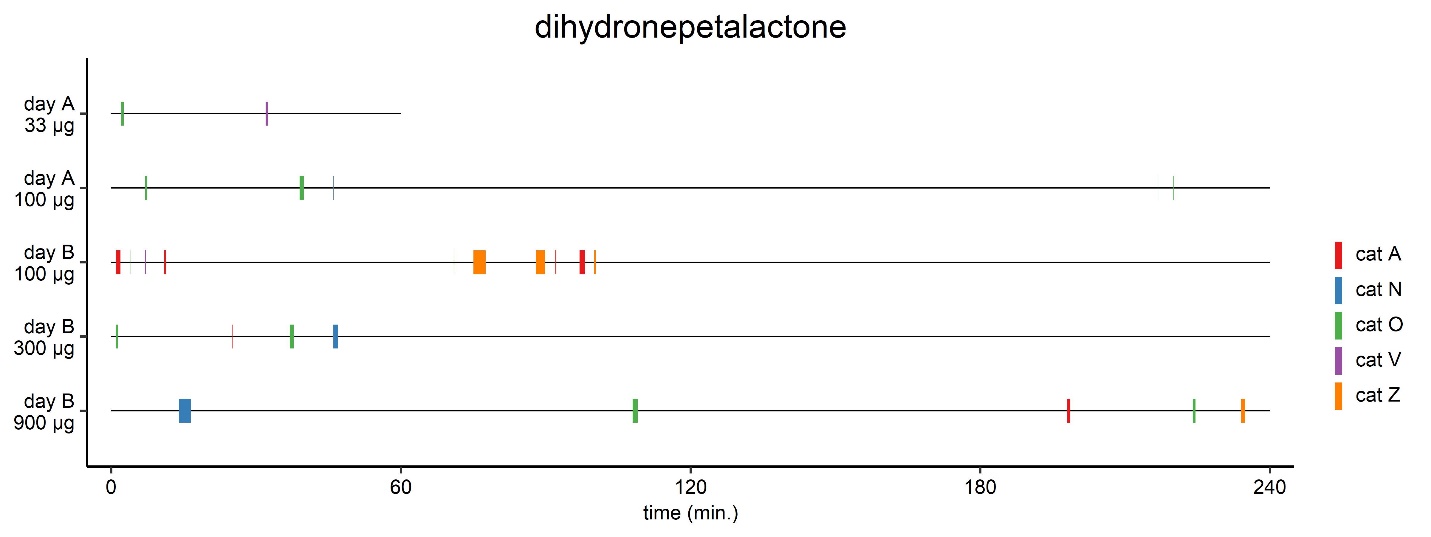

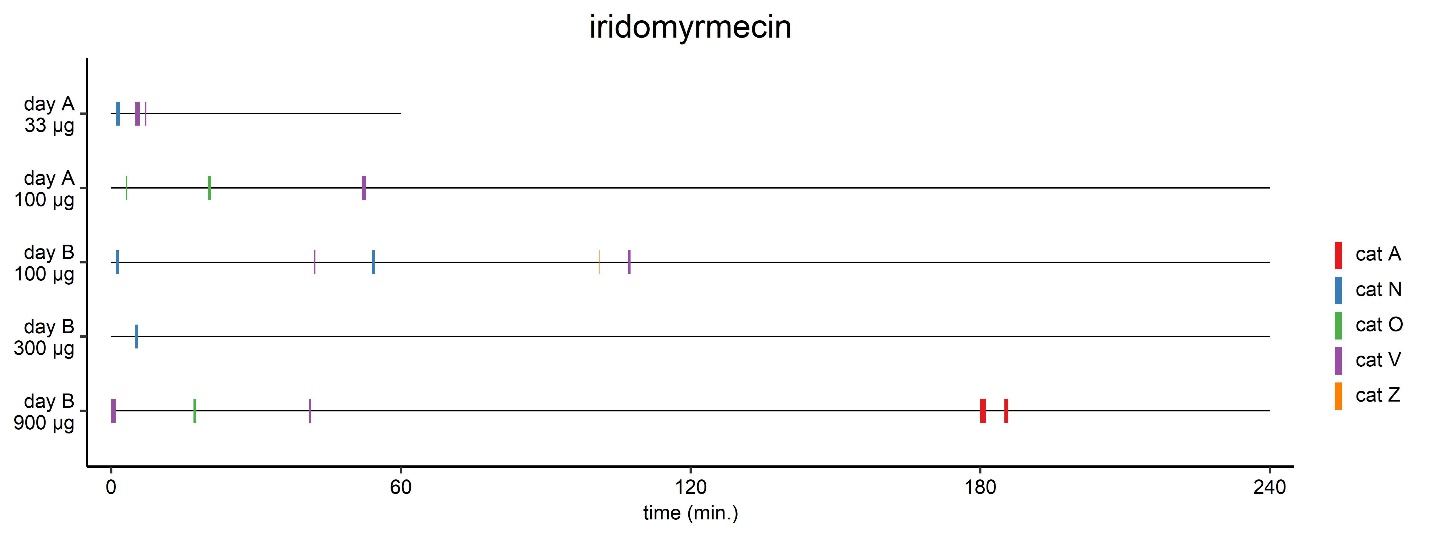

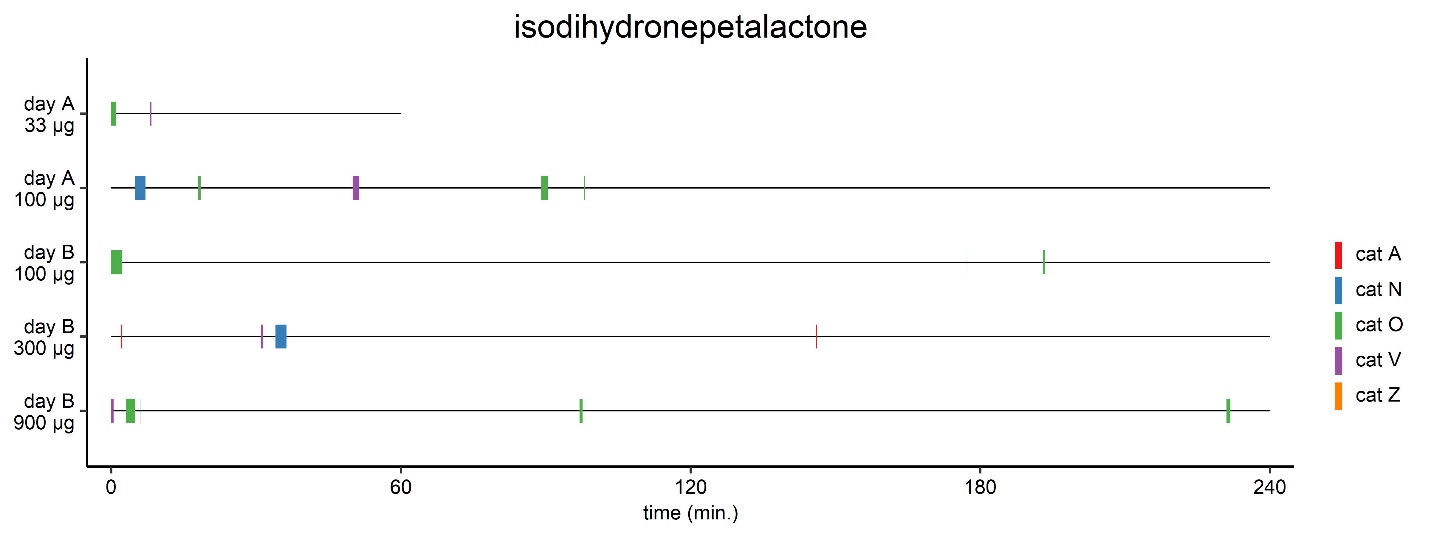

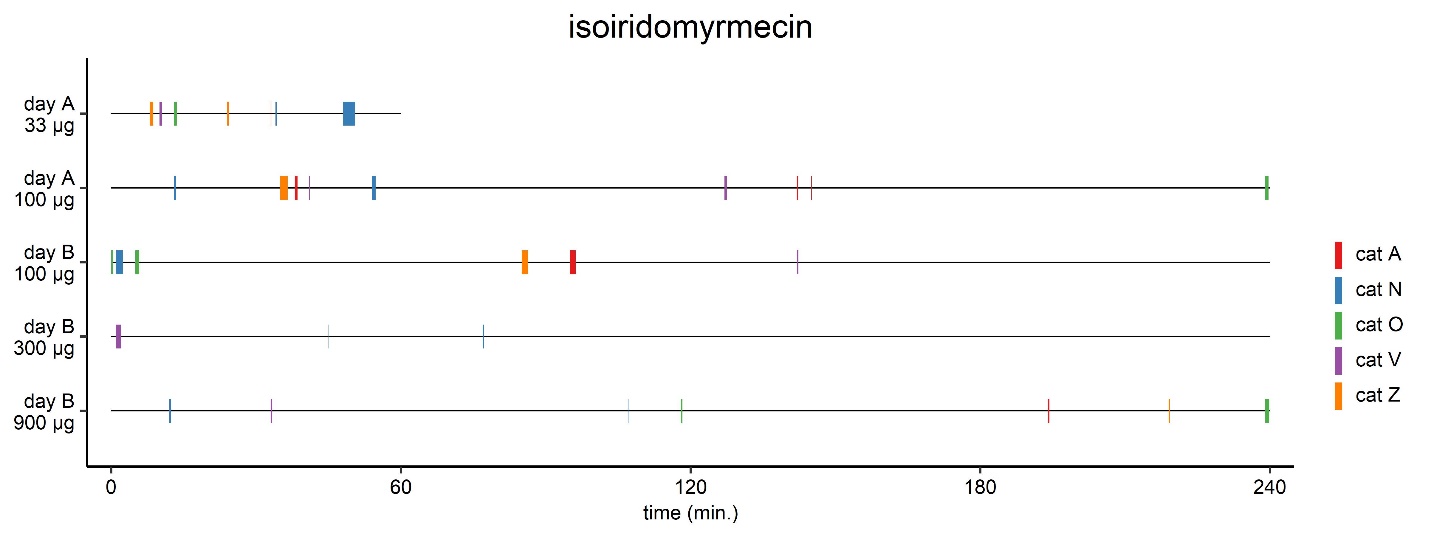

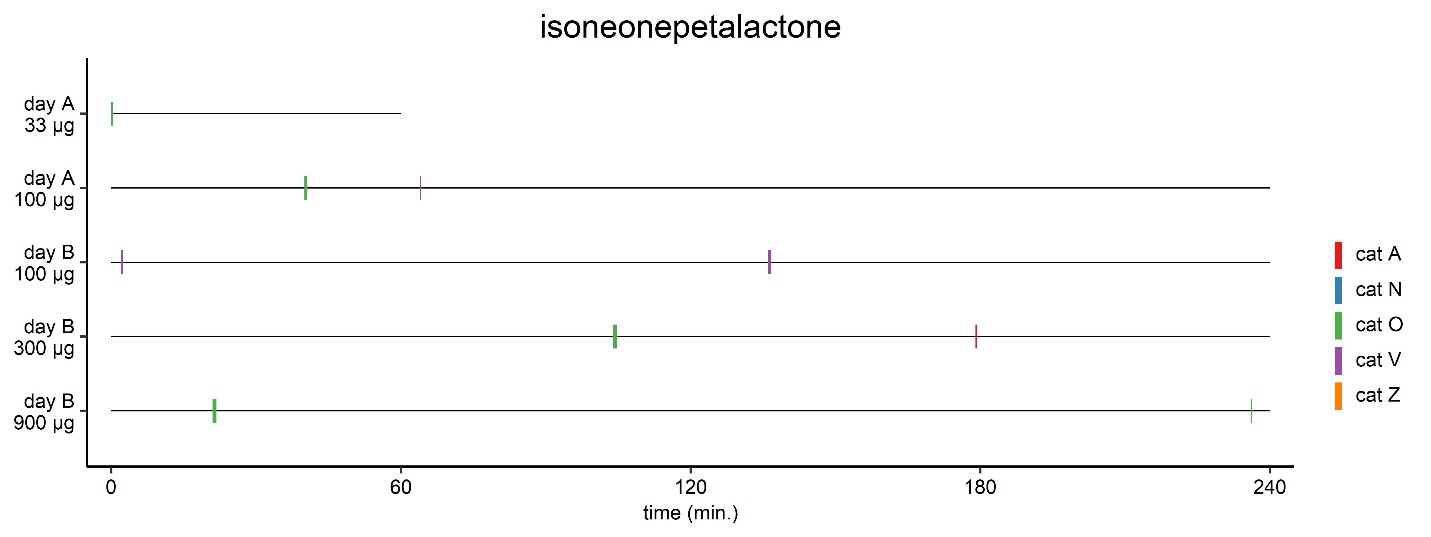

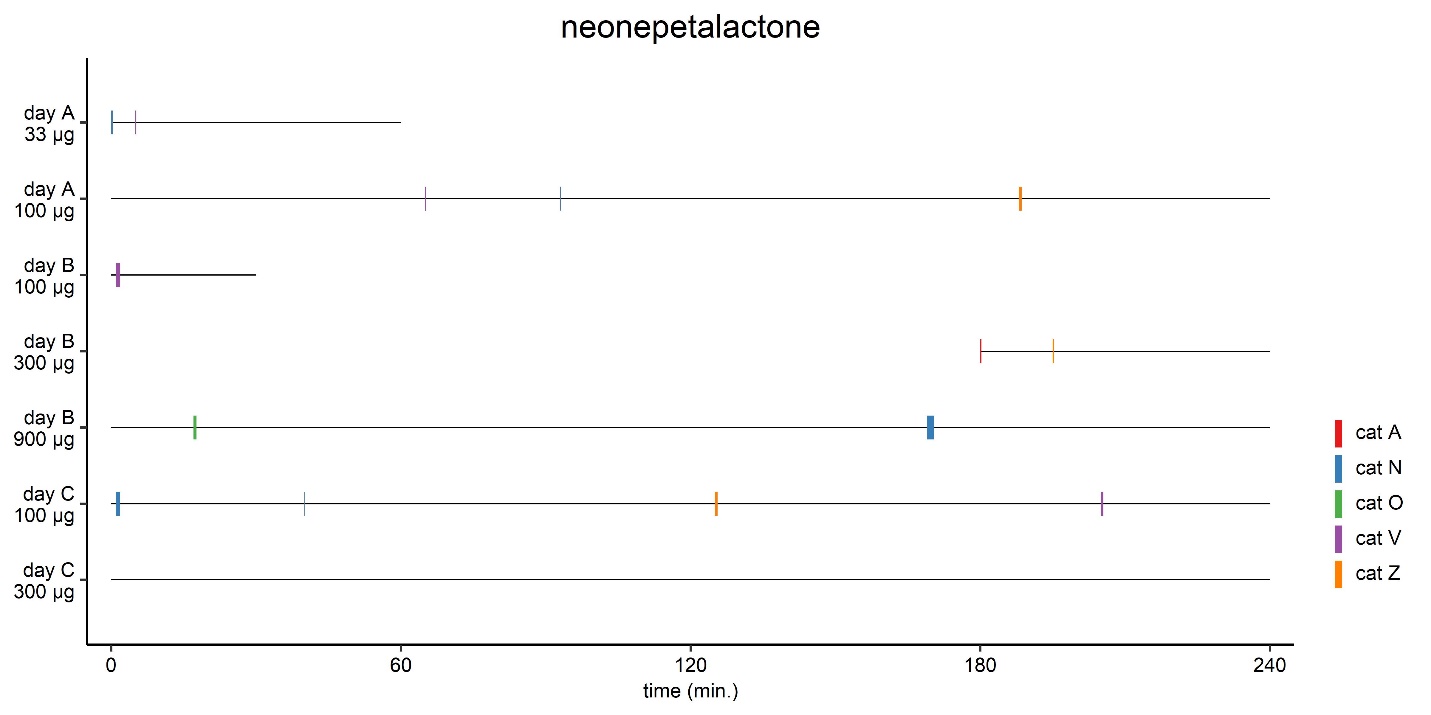

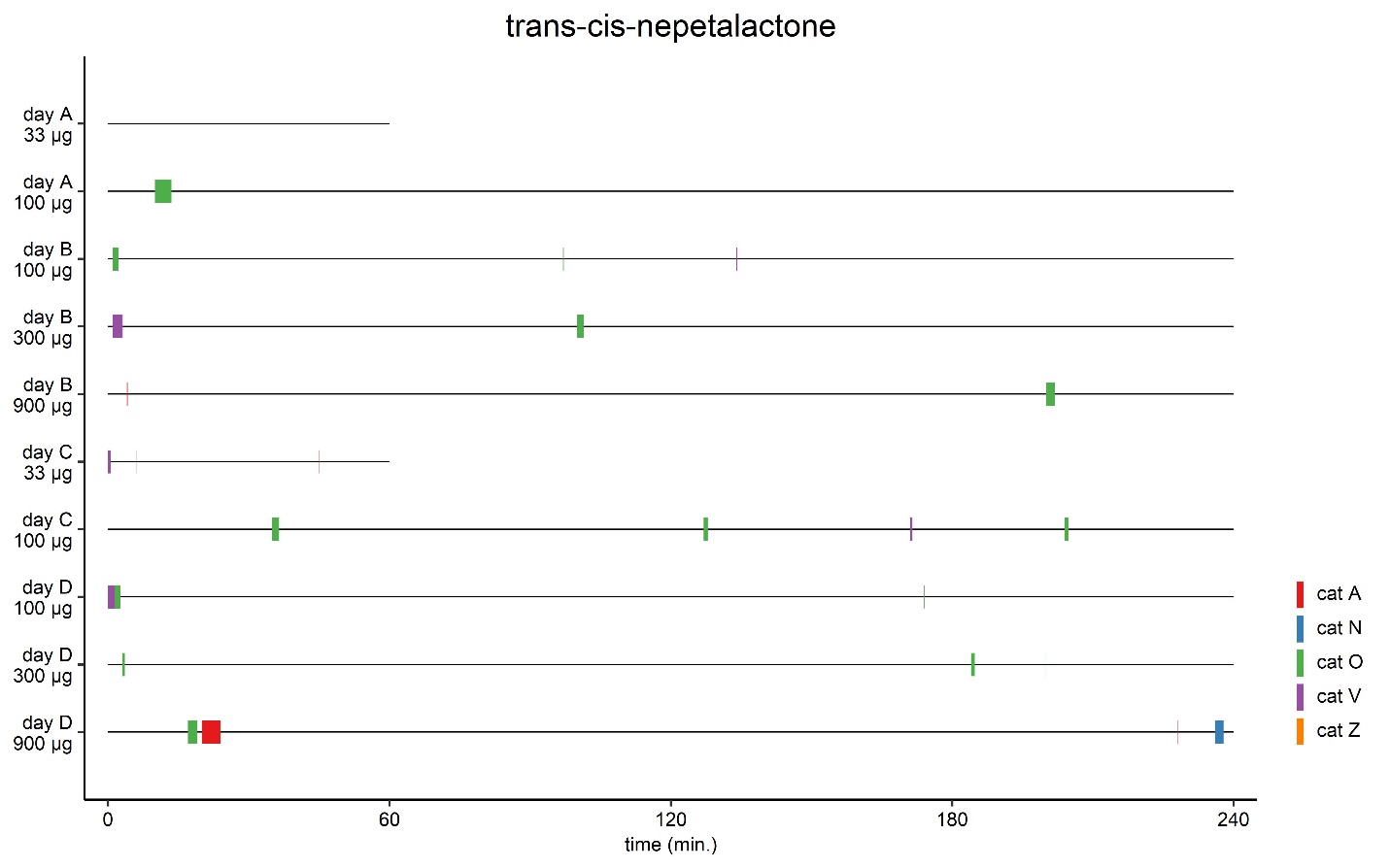


**Figure S7.** Graphical overview showing time of day of the responses, response frequency and response duration for the 5 domestic cats to the single compounds **1** – **10** (**Table 4**). Each compound was available on at least 2 days for a total of 17 hours. On the first day (day A), 33 and 100 µg were tested for 1 and 4 hours, respectively, typically in the afternoon. On the second day (day B), 100, 300 and 900 µg were tested, each for 4 hours, starting at 7:30, ending at 19:30. Because of the absence of response by some cats (**2** and **9**), technical problems (**5**), or testing a higher amount (2700 µg, **9**) some compounds were tested on additional days or for an extended period of time. Responses of only a couple of seconds sometimes do not show in the figure.


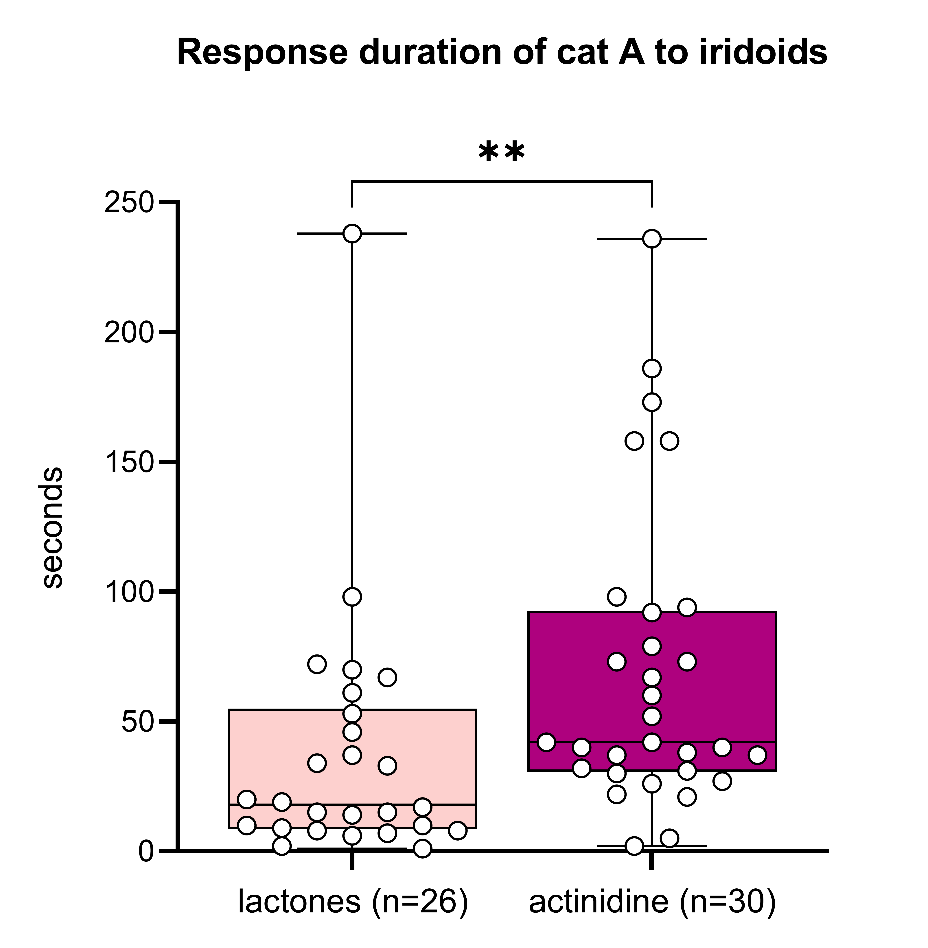


**Figure S8.** Duration of individual responses of cat A to the lactones and actinidine. Each individual response is shown as a dot. The difference in response duration between the lactones and actinidine is statistically significant (Mann-Whitney test). ** P < 0.01


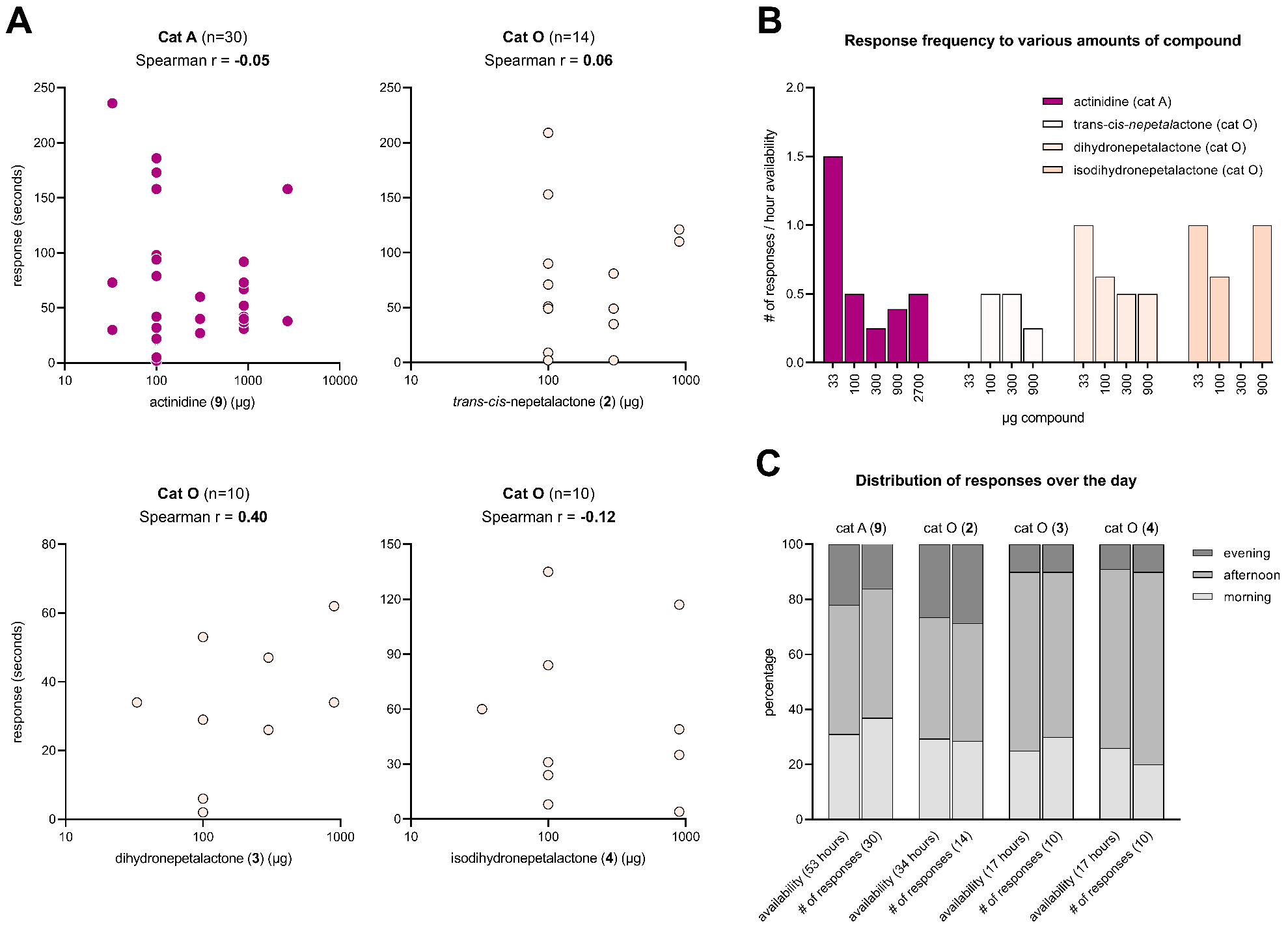


**Figure S9.** Data from compounds for which at least 10 responses from an individual cat were observed were used to study correlation between response duration/frequency and the amount of compound used, as well between response frequency and time of the day. (**A**) Response time to single compounds plotted against the various quantities of the compounds used in the tests: 33, 100, 300, 900 and 2700 µg. The quantities are shown on a log10 scale. (**B**) Response frequency per hour availability shown for the different quantities of single compounds tested. (**C**) Distribution of responses over the day (morning, afternoon and evening) compared to the distribution the olfactory stimuli were available to the cat (morning, afternoon and evening). Both the number of responses and the time each compound was available to the cat are expressed as a percentage. The total number of responses and hours availability are shown between parentheses. Bold numbers refer to the compound (**Table 4**). The Fisher exact test was used to test for differences in distribution (all P values > 0.05).


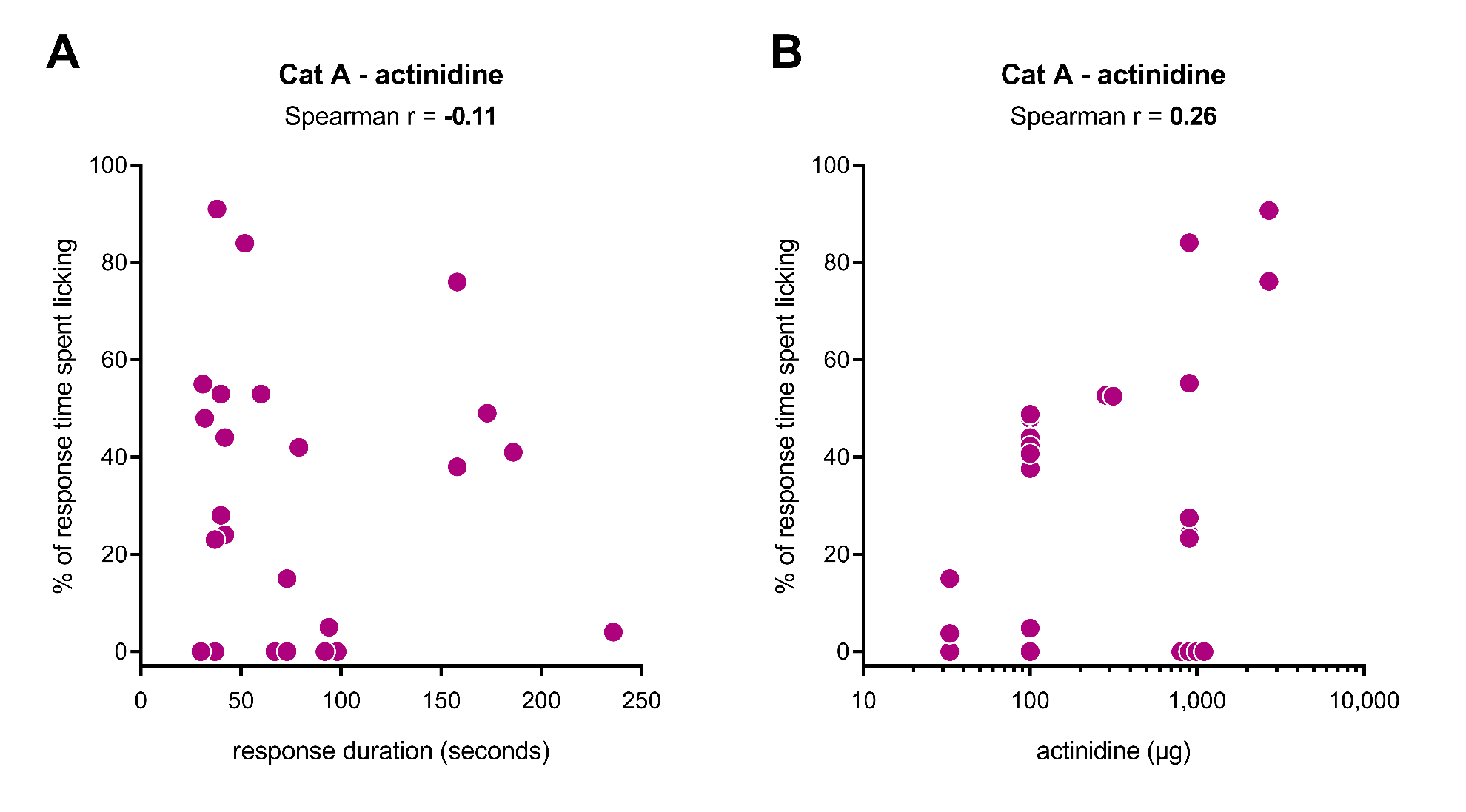


**Figure S10.** Absence of correlation between time spent licking and response duration (**A**) and time spent licking and actinidine quantity (33, 100, 300, 900 and 2700 µg) (**B**). Data from 24 responses longer than 30 seconds in duration to actinidine of cat A are shown. Some data points were overlapping and were edited for visualization purposes only. Actinidine quantity (µg) is shown on a log10 scale.


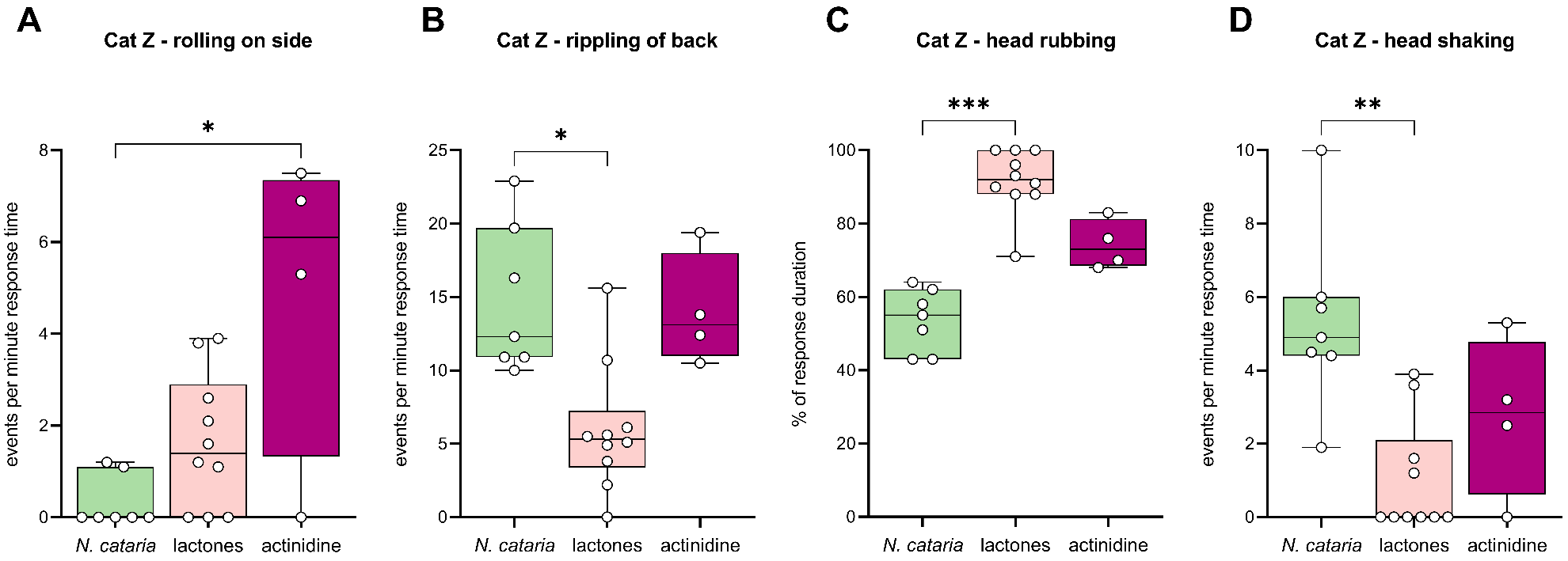


**Figure S11.** Differences in behavior of cat Z between responses to *N. cataria*, lactones and actinidine. * P < 0.05, ** P < 0.01, *** P < 0.001 (Dunn’s post-hoc test)


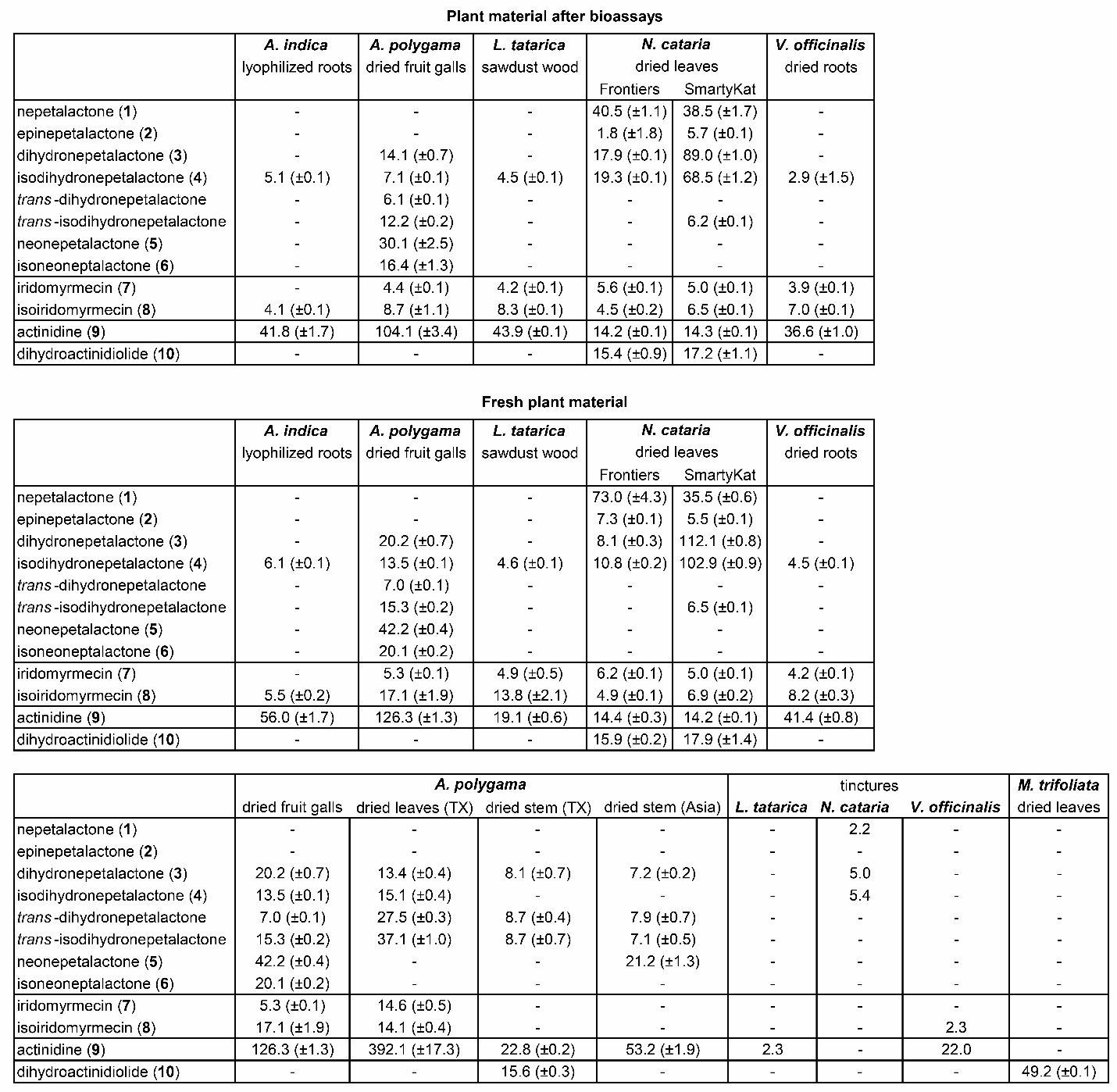


**Figure S12.** (**top**) Quantitation of cat-attracting compounds in the plant material taken from the socks after testing 10 × 10 hours in a 5-week testing period. Amounts are reported as µg per gram plant material used in this study. Reported values are the average of three separate extractions of the plant material. The standard error of the mean is reported between parentheses. Dashes indicate that the compound was not detected. (**middle and bottom**) Data from **Figure 21** reported with the standard error of the mean and unrounded numbers.


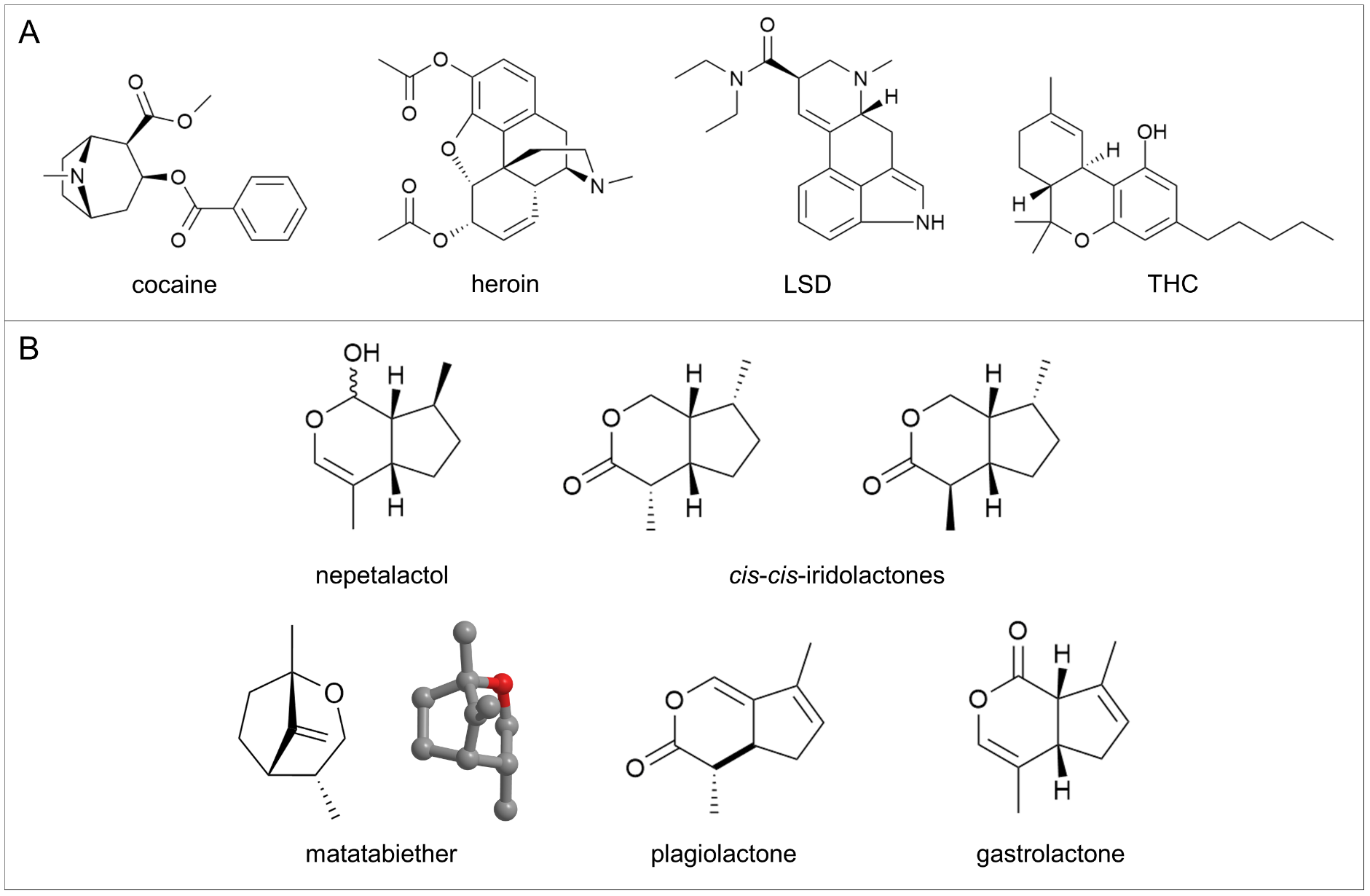


**Figure S13.** (**A**) The structures of cocaine, heroin, LSD and THC do not resemble the structure of the cat-attracting single compounds (**Figure 13**). (**B**) The structures of nepetalactol, both *cis*-*cis*-iridolactones, plagiolactone, gastrolactone, and both the 2D and 3D structure of the bridged bicyclic matatabiether.


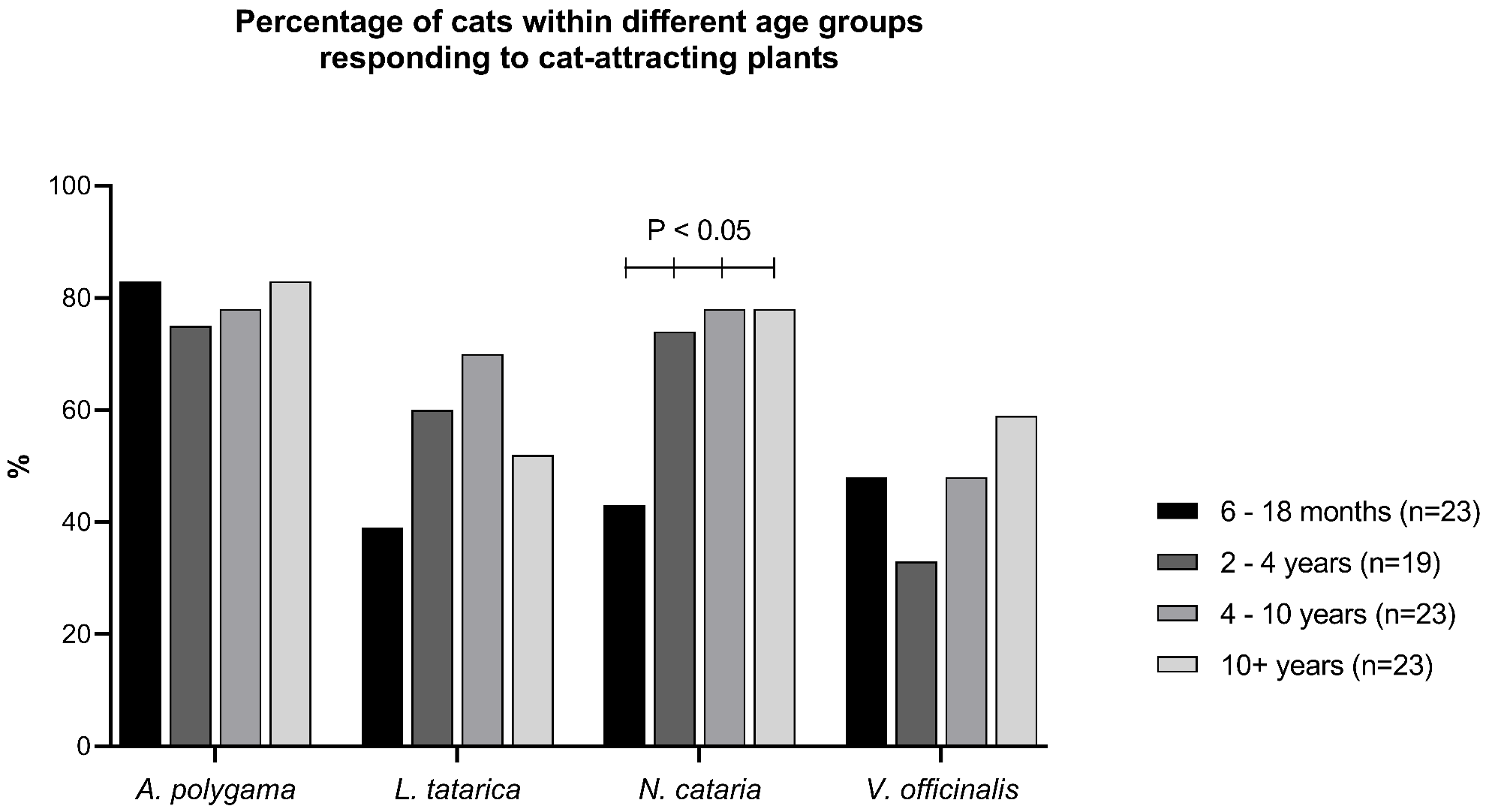


**Figure S14.** The fraction of domestic cats, within 4 age groups, that responded to a variety of cat-attracting plants. Significantly fewer of the younger cats (6 – 18 months) showed a response to catnip when compared to the 3 older groups (43% versus 74, 78 and 78%; P < 0.05, Fisher’s exact test). There were no cats 18 – 24 months of age.
